# Development of ProCaRS Clinical Nomograms for Biochemical Failure-free Survival Following Either Low-Dose Rate Brachytherapy or Conventionally Fractionated External Beam Radiation Therapy for Localized Prostate Cancer

**DOI:** 10.7759/cureus.276

**Published:** 2015-06-11

**Authors:** Andrew Warner, Tom Pickles, Juanita Crook, Andre-Guy Martin, Luis Souhami, Charles Catton, Himu Lukka, George Rodrigues

**Affiliations:** 1 Radiation Oncology, London Health Sciences Centre, London, Ontario, CA; 2 Radiation Oncology, BC Cancer Agency, Vancouver Centre, University of British Columbia; 3 Radiation Oncology, BC Cancer Agency, Kelowna, BC; 4 Radiation Oncology, Centre Hospitalier Universitaire de Québec - L'Hôtel-Dieu de Québec, Québec, QC; 5 Department of Oncology, Division of Radiation Oncology, McGill University Health Center; 6 Radiation Oncology, University of Toronto and Universitry Health Network; 7 Radiation Oncology, Juravinski Cancer Centre, Hamilton, ON; 8 Department of Oncology, London Health Sciences Centre; Schulich School of Medicine & Dentistry, Western University, London, Ontario, CA

**Keywords:** radiotherapy, prostate cancer, ldr brachytherapy, fractionated external beam radiation therapy, biochemical failure, nomogram

## Abstract

Purpose: Although several clinical nomograms predictive of biochemical failure-free survival (BFFS) for localized prostate cancer exist in the medical literature, making valid comparisons can be challenging due to variable definitions of biochemical failure, the disparate distribution of prognostic factors, and received treatments in patient populations. The aim of this investigation was to develop and validate clinically-based nomograms for 5-year BFFS using the ASTRO II “Phoenix” definition for two patient cohorts receiving low-dose rate (LDR) brachytherapy or conventionally fractionated external beam radiation therapy (EBRT) from a large Canadian multi-institutional database.

Methods and Materials: Patients were selected from the GUROC (Genitourinary Radiation Oncologists of Canada) Prostate Cancer Risk Stratification (ProCaRS) database if they received (1) LDR brachytherapy ≥ 144 Gy (n=4208) or (2) EBRT ≥ 70 Gy  (n=822). Multivariable Cox regression analysis for BFFS was performed separately for each cohort and used to generate clinical nomograms predictive of 5-year BFFS. Nomograms were validated using calibration plots of nomogram predicted probability versus observed probability via Kaplan-Meier estimates.

Results: Patients receiving LDR brachytherapy had a mean age of 64 ± 7 years, a mean baseline PSA of 6.3 ± 3.0 ng/mL, 75% had a Gleason 6, and 15% had a Gleason 7, whereas patients receiving EBRT had a mean age of 70 ± 6 years, a mean baseline PSA of 11.6 ± 10.7 ng/mL, 30% had a Gleason 6, 55% had a Gleason 7, and 14% had a Gleason 8-10. Nomograms for 5-year BFFS included age, use and duration of androgen deprivation therapy (ADT), baseline PSA, T stage, and Gleason score for LDR brachytherapy and an ADT (months), baseline PSA, Gleason score, and biological effective dose (Gy) for EBRT.

Conclusions: Clinical nomograms examining 5-year BFFS were developed for patients receiving either LDR brachytherapy or conventionally fractionated EBRT and may assist clinicians in predicting an outcome. Future work should be directed at examining the role of additional prognostic factors, comorbidities, and toxicity in predicting survival outcomes.

## Introduction

The treatment of localized prostate cancer in Canada has been evolving over the past few decades to reflect advances in our understanding of the disease and improvements in technology. Historically, single modality radiotherapy (low-dose rate (LDR) brachytherapy or external beam radiation therapy (EBRT)) has been utilized for patients with low and intermediate risk disease with optional androgen deprivation therapy (ADT) for select cases, whereas high-risk disease has typically been treated with EBRT (or less commonly high-dose-rate (HDR) brachytherapy, plus EBRT as dose escalation) followed by two to three years of adjuvant ADT [1-2]. Radical prostatectomy (RP) has typically been reserved for younger patients with clinically confined low-risk disease, and few or no comorbidities [2]. Minimal disease is increasingly being followed with active surveillance until progression [2].

Much of the existing literature to improve clinical decision making in prostate cancer management has been directed at the development and refinement of existing prognostic risk stratification systems and nomograms, often based on clinical endpoints (survival or biochemical) [3]. Collectively, risk stratification and nomograms in combination with other predictive modelling techniques, including regression tree analyses and artificial neural networks, assist in determining the appropriate treatment course by providing an assigned risk or survival probability prior to treatment selection [3]. Furthermore, several studies have demonstrated that nomograms can yield accurate models with predictability comparable to risk stratification models [4-8]. Many of the existing risk stratification systems are dominated by a set of three key prognostic factors – pre-treatment PSA, T stage, and Gleason score – each of which has been extensively validated to independently predict for a variety of clinical endpoints across a range of non-metastatic prostate cancer populations [3, 9]. 

Nomograms addressing biochemical and/or survival outcomes in prostate cancer have been published for patients receiving EBRT alone [4-5, 7, 10-17], one of RP or EBRT [18-20], RP or RP followed by salvage EBRT [21-28], and for LDR brachytherapy or LDR brachytherapy followed by EBRT [29-31], each reporting variable rates of ADT utilization. Similarly, the majority of published nomograms incorporate the same set of three key prognostic factors as reported in risk stratification systems (pre-treatment PSA, T stage and Gleason score), with some exceptions in studies reporting on RP or EBRT alone [10, 18] or RP with EBRT salvage [21, 23-28]. Other nomogram prognostic factors include: age [10, 15-16, 18, 24]; PSA doubling time [19, 27]; Gleason pattern [11, 28]; percentage of positive core biopsies [11, 15]; lymph node invasion [21, 23, 27-28]; ADT use or duration [4-5, 11-13, 15, 27]; EBRT use or dose (Gy) [4-5, 11-13, 15-16, 24-27, 30-31]; EBRT biological effective dose (BED) (Gy) [10]; and other RP-related features (positive surgical margin, RP type, extracapsular extension, and/or seminal vesicle invasion) [21-22, 26-28]. Nomograms have also been developed for similar patient populations examining non-survival-based endpoints such as: fecal incontinence [32-35]; rectal bleeding [34-35]; erectile function [36]; urinary retention [37-38]; and Gleason score upgrade [39-41] (Table 1).

**Table 1:** Published literature for nomograms based on external beam radiation therapy, LDR brachytherapy, and/or radical prostatectomy as primary or secondary treatments. EBRT – External Beam Radiation Therapy; LDR – Low-Dose Rate Brachytherapy; ADT – Androgen Deprivation Therapy; Sx – Surgery; BFFS – Biochemical Failure-Free Survival; BF – Biochemical Failure; CSM – Cancer-Specific Mortality; PFS – Progression-Free Survival; OS – Overall Survival; PSA – Prostate Specific Antigen

Author(s)	Year	Article Type	N	Primary Treatment	Secondary Treatment	ADT	Nomogram End Point(s)	Landmark Analysis	Landmark Threshold
Sanpaolo, et al.	2014	Observational	670	EBRT	No	Yes (70%)	6-year BFFS ("Phoenix")	No	N/A
Stoyanova, et al.	2013	Observational	2012	EBRT	No	Yes (22%)	8-year BFFS ("Phoenix")	No	N/A
Zelefsky, et al.	2011	Observational	2551	EBRT	No	Yes (49%)	5-, 10-year BFFS ("Phoenix")	No	N/A
Westphalen, et al.	2011	Observational	99	EBRT	No	Yes (N/A)	5-year BFFS ("Phoenix")	No	N/A
Spiess, et al.	2010	Observational	450	EBRT	Cryotherapy	Yes (21%)	BFFS ("PSA > 0.5 ng/mL")	No	N/A
Williams, et al.	2008	Observational	3264	EBRT	No	Yes (30%)	5-, 7-year BFFS ("Phoenix")	No	N/A
Zelefsky, et al.	2007	Observational	2253	EBRT	No	Yes (49%)	5-, 10-year BFFS ("Phoenix")	No	N/A
Williams, et al.	2006	Observational	1458	EBRT	No	No	5-, 7-year BFFS ("Phoenix")	No	N/A
Kattan, et al.	2003	Observational	1677	EBRT	No	Yes (7.6%)	5-year metastatic progression	No	N/A
Parker, et al.	2002	Observational	517	EBRT	No	Yes (100%)	1-year to 5-year BFFS ("2 consecutive PSA > 2 ng/mL")	No	N/A
Kattan, et al.	2000	Observational	1042	EBRT	No	Yes (37%)	5-year BFFS ("3 consecutive PSA rises")	No	N/A
Zelefsky, et al.	2012	Observational	1466	LDR	EBRT (36%)	Yes (31%)	5-year BFFS ("Phoenix")	No	N/A
Potters, et al.	2010	Observational	5931	LDR	EBRT (21%)	Yes (36%)	9-year BFFS ("Phoenix")	No	N/A
Kattan, et al.	2001	Observational	920	LDR	EBRT (18%)	No	5-year BFFS ("3 consecutive PSA rises")	No	N/A
Abdollah, et al.	2014	Observational	1107	Sx	EBRT (35%)	Yes (100%)	10-year CSM	No	N/A
Briganti, et al.	2013	Observational	472	Sx	EBRT	No	5-year BFFS ("2 consecutive PSA values ≥ 0.2 ng/mL")	No	N/A
Abdollah, et al.	2013	Observational	336	Sx	EBRT	Yes (42%)	10-year CSM	No	N/A
Porter, et al.	2010	Observational	752	Sx	EBRT (16%)	Yes (15%)	5-, 10-, 15-, 20-year CSM	No	N/A
Porter, et al.	2008	Observational	752	Sx	EBRT (16%)	Yes (6%)	Metastatic progression	No	N/A
Suardi, et al.	2008	Observational	601	Sx	EBRT (16%)	No	5-, 10-, 15-year BFFS ("PSA > 0.1 ng/mL")	No	N/A
Stephenson, et al.	2007	Observational	1540	Sx	EBRT	Yes (14%)	6-year PFS	No	N/A
Stephenson, et al.	2005	Observational	1881	Sx	EBRT (1%)	No	10-year PFS	No	N/A
Walz, et al.	2007	Observational	9131	Sx or EBRT	No	No	10-year OS	No	N/A
Slovin, et al.	2005	Other	148	Sx or EBRT	No	No	1-, 2-year PFS, Median PFS	No	N/A
D'Amico, et al.	1999	Observational	1654	Sx or EBRT	No	No	2-year BFFS ("3 consecutive PSA rises")	No	N/A
Fellin, et al.	2014	Observational	515	EBRT	No	Yes (89%)	Grade 1-3 late fecal incontinence	No	N/A
Chipman, et al.	2014	Observational	1201	Sx, EBRT or LDR	No	Yes (N/A)	2-Year functional erection	No	N/A
Mathieu, et al.	2014	Observational	965	EBRT	No	Yes (23%)	5-year grade 2-4 urinary toxicity	No	N/A
Bowes, et al.	2012	Observational	259	LDR-Brachy	No	No	Gleason score upgrade	No	N/A
Fiorino, et al.	2012	Other	586	EBRT	No	No	Late fecal incontinence	No	N/A
Valdagni, et al.	2012	Other	718	EBRT	No	Yes (78%)	Grade 2-3 late rectal bleeding, fecal incontinence	No	N/A
Roeloffzen, et al.	2011	Observational	714	LDR-Brachy	No	Yes (19%)	Acute urinary retention	Yes	6 months
Budäus, et al.	2010	Observational	414	Sx	No	No	Gleason score upgrade	No	N/A
Valdagni, et al.	2008	Other	1124	EBRT	No	Yes (74%)	Grade 2-3 acute lower GI toxicity, moderate/severe stool frequency, severe incontinence, moderate/severe acute rectal bleeding	No	N/A
Kulkarni, et al.	2007	Observational	175	Sx	No	No	Gleason score upgrade	No	N/A
Kattan, et al.	2003	Observational	409	Sx	No	No	Indolent cancer (< 0.5 cc)	No	N/A

Comparisons between existing nomograms are limited due to heterogeneity in reported clinical endpoints, in the proportion of patients receiving secondary/salvage EBRT, and in the proportion/duration/timing of ADT use. Additionally, variability exists between individual patient populations in terms of the distribution of known and unknown prognostic factors (confounders) directly impacting the degree of generalizability between patient populations. Specifically, nomograms developed using biochemical failure definitions, which differ from the ASTRO II “Phoenix” biochemical failure-free survival 2006 consensus definition of a PSA increase of 2 ng/mL above the nadir PSA, are further limited in the ability to make direct comparisons [42]. The overall aim of this investigation was to develop and validate clinically-based nomograms for 5-year biochemical failure-free survival using the landmark method separately for two patient cohorts receiving LDR brachytherapy or conventionally fractionated EBRT in the context of a large Canadian multi-institutional prostate radiotherapy database.

## Materials and methods

### The GUROC ProCaRS database

The GUROC (Genitourinary Radiation Oncologists of Canada) Prostate Cancer Risk Stratification (ProCaRS) database was created by combining retrospectively collected data from 7,974 patients with localized prostate cancer treated with primary LDR brachytherapy, HDR brachytherapy, or conventionally fractionated EBRT (or combination). Patients receiving RP as the primary treatment were not included in the database. All patients were treated between 1994 and 2010 at one of four participating Canadian institutions (British Columbia Cancer Agency (n=3,771), Princess Margaret Hospital (n=1,752), McGill University Health Centre (n=194), and L’Hotel Dieu de Québec (n=2,257)). Further details pertaining to the assembly and quality assurance procedures for the GUROC ProCaRS database have been described previously [43-44].

### Patient selection

**Figure 1:**
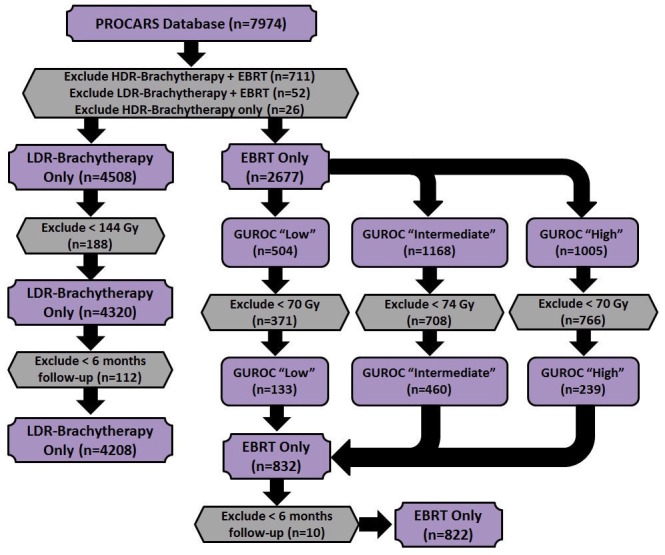
Summary of patient selection and creation of final analysis cohorts (LDR – Low-Dose Rate (Brachytherapy), HDR – High-Dose Rate (Brachytherapy), EBRT – External Beam Radiation Therapy).

Patients receiving LDR brachytherapy ≥ 144 Gy alone (n=4,320) or EBRT (n=832) were eligible for analysis. To address EBRT dose heterogeneity across participating institutions and to ensure the nomograms would be relevant to modern radiotherapy practice, only patients identified as receiving sufficient dose escalation, using either three-dimensional conformal therapy or intensity-modulated radiation therapy (defined as ≥ 70 Gy (GUROC low-risk and high-risk) or ≥ 74 Gy (GUROC intermediate risk)) were considered for this analysis [45]. To reduce the risk of survival bias, landmark analysis techniques were applied by manually excluding patients with follow-up durations below an a priori selected landmark threshold [46-49]. Generally, the use of landmark analysis in the development of nomograms for prostate cancer has been absent with the exception of a study published by Roeloffzen, et al. in 2011 examining acute urinary retention in patients receiving LDR brachytherapy [38]. Six months was selected for the present study in order to maintain sufficient statistical power for analysis relative to the primary endpoint of 5-year biochemical failure-free survival [47-48]. Therefore, patients with follow-up durations less than six months were excluded from analysis (LDR brachytherapy (n=112); EBRT (n=10)). This formed two final analysis cohorts of 4,208 patients receiving LDR brachytherapy and 822 patients receiving EBRT. Details of patient selection are shown in Figure 1.

### Endpoints

The primary endpoint was ASTRO II “Phoenix” biochemical failure-free survival (BFFS) defined as time from initiation of radiotherapy to the date of last follow-up and/or biochemical failure, whichever came first, according to the definition of a PSA increase of 2 ng/mL above the nadir PSA [42]. Technical biochemical failures arising from benign PSA bounces were adjusted using a quality assurance procedure reported previously and applied to both treatment cohorts [43, 45, 50]. Patients with PSA levels that returned to an absolute level of ≤ 0.5 ng/mL without intervention were re-classified as not to have had a biochemical failure, whereas patients with PSA levels > 0.5 ng/mL following biochemical failure were still considered to have biochemically failed [43, 45, 50]. Nomogram predicted individual patient-level probability estimates of 5-year BFFS were also obtained based on multivariable Cox proportional hazards regression for BFFS.  Although overall survival (OS) and cancer-specific survival were available in the GUROC ProCaRS database, these were not examined due to an insufficient number of events and/or limited available follow-up required for multivariable regression and nomogram analysis for meaningful nomogram construction.

### Statistical analysis

Descriptive statistics were generated for baseline patient, tumour and treatment characteristics for all patients (n=5,030) and stratified by treatment cohort (LDR brachytherapy only (n=4,208), EBRT only (n=822)). Univariable Cox proportional hazards regression analysis was performed on a set of prognostic factors previously shown to be significant predictors of BFFS and/or OS, based on the complete GUROC ProCaRS database, to identify significant predictors of ASTRO II “Phoenix” BFFS separately by treatment cohort. This list included age, ADT (yes/no and duration), baseline PSA (ng/mL), T stage, Gleason score (categorical), and positive core percentage [43-44]. BED defined with a α/β of 2, was additionally examined in the EBRT cohort.  Multivariable Cox proportional hazards regression was performed for BFFS separately by treatment cohort using backward elimination techniques, beginning with all eligible factors and sequentially removing factors until all remaining covariates had p-values < 0.20. Given the high degree of missing data for positive core percentage, this specific variable was only reported in univariable procedures.

Nomograms based on the final multivariable Cox proportional hazards regression models for BFFS were generated separately by treatment cohort to calculate individual patient-level probability estimates for 5-year BFFS, according to each patient’s particular combination of baseline characteristics. Nomogram validation was performed via calibration plots of observed probability (calculated using Kaplan-Meier estimates) by nomogram predicted probability for 5-year BFFS for each nomogram separately. All statistical analysis was performed using SAS version 9.3 software (SAS Institute, Cary, NC) and the R language environment for statistical computing version 3.0.3, using two-sided statistical testing at the 0.05 significance level. 

## Results

Baseline patient characteristics are presented in Table 2. Overall for patients receiving LDR brachytherapy: mean age was 64 ± 7 years; mean baseline PSA was 6.3 ± 3.0 ng/mL; 55% and 45% had T1 and T2 disease, respectively; 75% had Gleason 6 and 15% had Gleason 7; 27% had ≥ 50% positive core biopsies; 38% received ADT (36% for ≥ 6 months; 3.7% for ≥ 1 year); and 75%, 24%, and 1% of patients were classified as GUROC low-, intermediate-, and high-risk, respectively. Biochemical failure was observed in 216 patients (5%), 168 (4%) occurring within five years of radiotherapy, and patient deaths were observed in 239 (6%) (138 (3%) within five years of radiotherapy). The median actuarial follow-up was 5.55 years. For patients receiving EBRT: mean age was 70 ± 6 years; mean baseline PSA was 11.6 ± 10.7 ng/mL; 36% and 50% had T1 and T2 disease, respectively; 30% had Gleason 6, 55% had Gleason 7, and 14% had Gleason 8-10; 50% had ≥ 50% positive core biopsies; 49% received ADT (18% for ≥ 2 years; 8% for ≥ 3 years); and 16%, 55%, and 29% were classified as GUROC low-, intermediate-, and high-risk, respectively.  Biochemical failure was observed in 228 patients (28%), 147 (18%) occurring within five years of radiotherapy, and patient deaths were observed in 113 (14%) (49 (6%) occurring within five years of radiotherapy). The median actuarial follow-up was 7.10 years.

**Table 2:** Baseline tumour, patient and treatment characteristics for: (A) all patients (n=5030), (B) LDR Brachytherapy only (n=4208), and (C) EBRT only (n=822). PSA – Prostate Specific Antigen; LDR – Low-Dose Rate Brachytherapy; EBRT – External Beam Radiation Therapy; ADT – Androgen Deprivation Therapy

Characteristic	N	All Patients (n=5030)	LDR Only (n=4208)	EBRT Only (n=822)
Centre – n(%)				
British Columbia Cancer Agency	5030	2098 (41.7)	1757 (41.8)	341 (41.5)
Princess Margaret Hospital		1399 (27.8)	918 (21.8)	481 (58.5)
L’Hotel Dieu de Québec		1533 (30.5)	1533 (36.4)	--
Age – mean ± SD, median, (min, max)	5029	65.31 ± 7.20	64.35 ± 7.03	70.23 ± 5.99
		66.00	65.00	71.00
		(34.00, 84.00)	(40.00, 83.00)	(34.00, 84.00)
Baseline PSA (ng/mL) – mean ± SD, median, (min, max)	4958	7.19 ± 5.52	6.31 ± 2.99	11.61 ± 10.74
		6.20	5.98	8.47
		(0.10, 130.75)	(0.10, 40.00)	(0.26, 130.75)
T stage – n(%)				
T1	4951	2573 (52.0)	2284 (55.2)	289 (35.5)
T2		2255 (45.6)	1849 (44.7)	406 (49.9)
T3		117 (2.4)	4 (0.1)	113 (13.9)
T4		6 (0.1)	--	6 (0.7)
Gleason score – n(%)				
2-5	4958	400 (8.1)	382 (9.2)	18 (2.2)
6		3362 (67.8)	3119 (75.4)	243 (29.6)
7		1075 (21.7)	627 (15.2)	448 (54.6)
8-10		121 (2.4)	9 (0.2)	112 (13.6)
Positive vores (%) – mean ± SD, median, (min, max)	3036	37.07 ± 23.02	33.96 ± 20.74	48.10 ± 26.99
		33.33	30.00	45.64
		(5.26, 100.00)	(5.26, 100.00)	(5.56, 100.00)
Radiotherapy treatment year – n(%)				
1994-1999	5030	439 (8.7)	396 (9.4)	43 (5.2)
2000-2002		1520 (30.2)	1136 (27.0)	384 (46.7)
2003-2005		1894 (37.7)	1535 (36.5)	359 (43.7)
2006-2010		1177 (23.4)	1141 (27.1)	36 (4.4)
EBRT: Dose (Gy) – mean ± SD, median, (min, max)	822	76.37 ± 3.65	--	76.37 ± 3.65
		75.60	--	75.60
		(70.00, 79.80)	--	(70.00, 79.80)
EBRT: Number of fractions – mean ± SD, median, (min, max)	822	39.61 ± 2.89	--	39.61 ± 2.89
		42	--	42
		(35, 42)	--	(35, 42)
EBRT: Dose per fraction (Gy) – mean ± SD, median, (min, max)	822	1.93 ± 0.07	--	1.93 ± 0.07
		1.90	--	1.90
		(1.80, 2.11)	--	(1.80, 2.11)
EBRT: Biological effective dose (Gy)– mean ± SD, median, (min, max)	822	150.08 ± 5.95	--	150.08 ± 5.95
		148.00	--	148.00
		(136.80, 156.00)	--	(136.80, 156.00)
ADT – n(%)	5030	1995 (39.7)	1589 (37.8)	406 (49.4)
ADT (months) – mean ± SD, median, (min, max)	1738	7.89 ± 8.13	6.08 ± 4.99	13.89 ± 12.52
		5.88	5.75	9.33
		(0.30, 132.67)	(0.30, 132.67)	(0.49, 99.68)
Pathologically confirmed local relapse – n(%)	5030	107 (2.1)	31 (0.7)	76 (9.3)
ASTRO II “Phoenix” biochemical bailure – n(%)	4850	444 (9.2)	216 (5.3)	228 (28.4)
5-year ASTRO II “Phoenix” biochemical failure – n(%)	4850	315 (6.5)	168 (4.2)	147 (18.3)
Death – n(%)	5030	352 (7.0)	239 (5.7)	113 (13.8)
5-year death – n(%)	5030	187 (3.7)	138 (3.3)	49 (6.0)
Prostate cancer death – n(%)	5030	51 (1.0)	24 (0.6)	27 (3.3)
5-year prostate cancer death – n(%)	5030	30 (0.6)	17 (0.4)	13 (1.6)
Cause of death – n(%)				
Dead of disease	352	51 (14.5)	24 (10.0)	27 (23.9)
Dead (other)		223 (63.4)	160 (67.0)	63 (55.8)
Dead (NOS)		78 (22.2)	55 (23.0)	23 (20.4)
GUROC – n(%)				
Low	4969	3253 (65.5)	3122 (75.3)	131 (15.9)
Intermediate		1451 (29.2)	999 (24.1)	452 (55.0)
High		265 (5.3)	26 (0.6)	239 (29.1)
ProCaRS 5 – n(%)				
Low	4940	3253 (65.9)	3122 (75.8)	131 (16.0)
Low-intermediate		1265 (25.6)	913 (22.2)	352 (42.9)
High-intermediate		162 (3.3)	58 (1.4)	104 (12.7)
High		192 (3.9)	21 (0.5)	171 (20.8)
Very high		68 (1.4)	5 (0.1)	63 (7.7)
Actuarial follow-up (years) (using reverse Kaplan-Meier method) –median (min, max)	5030	5.81	5.55	7.10
		(0.50, 15.15)	(0.50, 15.15)	(0.54, 11.45)

### Cox proportional hazards regression

Results from univariable Cox proportional hazards regression for BFFS are shown in Table 3. For LDR brachytherapy, only ADT duration (hazard ratio [HR] per one month increase: 1.03; 95% CI: 1.01-1.05, p=0.008) and baseline PSA (HR per 1 ng/mL increase: 1.10; 95% CI: 1.07-1.14, p<0.001) were significant independent predictors of BFFS. Multivariable modelling also identified ADT duration (HR: 1.03; 95% CI: 1.02-1.05, p<0.001) and baseline PSA (HR: 1.11; 95% CI: 1.08-1.15, p<0.001) as significant predictors in addition to age (HR per one year increase: 0.98; 95% CI: 0.96-1.00, p=0.038) and receiving ADT (HR: 0.53; 95% CI: 0.37-0.76, p<0.001). T stage (p=0.164) and Gleason score (p=0.080) also met the criteria for inclusion in the final multivariable regression model (p<0.20) but were not found to be statistically significant. For EBRT, baseline PSA (HR per 1 ng/mL increase: 1.02; 95% CI: 1.01-1.03, p<0.001), T stage (p=0.047), Gleason score (p=0.002), BED (HR per 10 Gy increase: 0.81; 95% CI: 0.66-0.99, p=0.043), and positive core percentage (HR per 1 percent increase: 1.01; 95% CI: 1.01-1.02, p<0.001) were significant independent predictors of BFFS. From multivariable modelling, only baseline PSA (HR: 1.02; 95% CI: 1.01-1.03, p<0.001) and Gleason score (p=0.005) remained significant with ADT duration (p=0.092) and BED (p=0.138) remaining eligible for inclusion in the final model (p<0.20) but were not statistically significant.

**Table 3:** Univariable and multivariable Cox regression models of factors predicting ASTRO II “Phoenix” Biochemical Failure-Free Survival for (A) LDR Brachytherapy only (n=4208) and (B) EBRT only (n=822). PSA – Prostate Specific Antigen; ADT – Androgen Deprivation Therapy; HR – Hazard Ratio; CI – Confidence Interval; P-values < 0.05 shown as BOLD, **overall analysis of effects (applicable to categorical variables only).

Dependent Variable:	ASTRO II “Phoenix” Biochemical Failure-Free Survival			
(A) LDR Brachytherapy only (n=4208)	Univariable		Multivariable	
Independent Variables:	HR (95% CI)	p-value	HR (95% CI)	p-value
Age	0.99	0.218	0.98	0.038
Per 1 year increase	(0.97, 1.01)		(0.96, 1.00)	
ADT	0.93	0.581	0.53	< 0.001
Yes vs No	(0.70, 1.22)		(0.37, 0.76)	
ADT duration	1.03	0.008	1.03	< 0.001
Per 1-month increase	(1.01, 1.05)		(1.02, 1.05)	
Baseline PSA	1.10	< 0.001	1.11	< 0.001
Pper 1 ng/mL increase	(1.07, 1.14)		(1.08, 1.15)	
T stage	1.25	0.101	1.22	0.164
T2 or T3 vs T1	(0.96, 1.64)		(0.92, 1.62)	
Gleason score		**0.218		**0.080
6 vs 2-5	1.01	0.948	1.11	0.657
	(0.67, 1.54)		(0.71, 1.72)	
7-10 vs 2-5	1.38	0.203	1.68	0.052
	(0.84, 2.28)		(1.00, 2.82)	
Positive cores percentage	1.00	0.467	--	--
Per 1 percent increase	(1.00, 1.01)		--	
(B) EBRT only (n=822)	Univariable		Multivariable	
Independent variables:	HR (95% CI)	p-value	HR (95% CI)	p-value
Age	0.99	0.617	--	--
Per 1 year increase	(0.97, 1.02)		--	
ADT	1.12	0.381	--	--
Yes vs No	(0.87, 1.46)		--	
ADT duration	1.00	0.619	0.99	0.092
Per 1 month increase	(0.99, 1.01)		(0.97, 1.00)	
Baseline PSA	1.02	< 0.001	1.02	< 0.001
Per 1 ng/mL increase	(1.01, 1.03)		(1.01, 1.03)	
T stage		**0.047		*--*
2 vs 1	1.48	0.010	--	*--*
	(1.10, 1.99)		--	
3 vs 1	1.53	0.062	--	*--*
	(0.98, 2.40)		--	
4 vs 1	2.11	0.208	--	*--*
	(0.66, 6.70)		--	
Gleason score		**0.002		**0.005
7 vs 2-6	1.52	0.011	1.53	0.010
	(1.10, 2.09)		(1.11, 2.10)	
8-10 vs 2-6	2.05	< 0.001	2.01	0.003
	(1.36, 3.11)		(1.28, 3.17)	
EBRT biological effective dose	0.81	0.043	0.98	0.138
Per 10 Gy increase	(0.66, 0.99)		(0.96, 1.01)	
Positive cores percentage	1.01	< 0.001	--	--
Per 1 percent increase	(1.01, 1.02)		--	

### Clinical nomograms 

**Figure 2:**
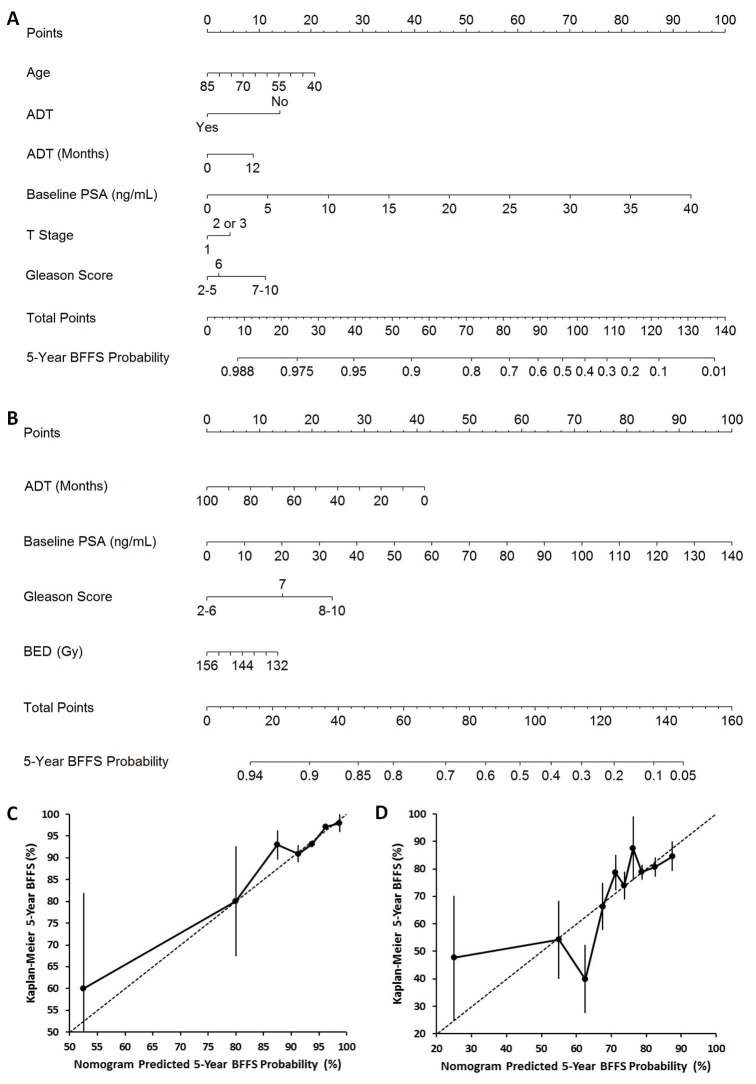
Nomograms and corresponding calibration plots predicting 5-year ASTRO II “Phoenix” Biochemical Failure-Free Survival for (A,C) LDR Brachytherapy only (n=4208) and (B,D) EBRT only (n=822).

Prognostic factors identified from the multivariable Cox regression model for BFFS for the LDR brachytherapy cohort are depicted in a nomogram shown in Figure 2A. Based on the calibration plot shown in Figure 2C, the nomogram showed reasonable calibration with only minimal underestimation of the true BFFS percentages for patients with nomogram-predicted probabilities below approximately 70% and approaching 90%. The deviation in the calibration plot below approximately 70% can be partially attributed to the limited number of patients with observed worse BFFS available for testing. Factors shown to be predictive of BFFS for the EBRT cohort from multivariable Cox regression were entered into the nomogram shown in Figure 2B. The calibration plot shown in Figure 2D demonstrated reasonable calibration for nomogram-predicted probabilities above 70%, whereas, below 70%, a combination of underestimation and overestimation of true BFFS was observed. Similarly, deviation in the calibration plot at the lower extreme can be partially attributed to the limited number of patients with observed worse BFFS available for testing.

## Discussion

This investigation presented a comprehensive set of clinical nomograms to predict for 5-year BFFS separately for patients receiving LDR brachytherapy and conventionally fractionated EBRT based on a large multi-institutional database. It additionally presented a systematic data-driven approach to the development of each nomogram incorporating only those factors shown to be associated with BFFS in the examined database. For LDR brachytherapy, age, ADT duration, and baseline PSA emerged as important predictors of BFFS from both univariable and multivariable analysis and, to a lesser degree, whether or not patients received ADT and Gleason score. For EBRT, baseline PSA, T stage, Gleason score, BED, and positive core percentage were significant independent predictors of BFFS, with particular importance observed for baseline PSA and Gleason score being significant from multivariable analysis.

The importance of key prognostic factors established in the literature (pre-treatment PSA, T stage, and Gleason score) was also demonstrated in the current study. However, this effect was prevalent more for pre-treatment PSA and Gleason score, with both variables being represented in each nomogram. T stage was only incorporated into the nomogram for the LDR brachytherapy cohort based on the a priori specified criteria for inclusion in multivariable regression models (p<0.20). Upon further examination, T stage was shown to be an independent significant predictor of BFFS (p=0.047) for the EBRT cohort but was only somewhat associated with BFFS for the LDR brachytherapy cohort (p=0.101). Similar nomograms predictive of either BFFS or OS have also been published omitting T stage [10, 18, 23, 27-28]. In contrast, pre-treatment PSA, T stage, and Gleason score were shown to be predictive of BFFS from multivariable regression reported for the entire GUROC ProCaRS database, which pooled data from patients receiving a variety of radiotherapy approaches [44]. This suggests that the process of creating more homogeneous cohorts of patients by restricting to only one type of radiotherapy and range of delivered dose, in addition to incorporating landmark analysis techniques, may be the primary contributing factor to explaining the observed differences. 

Additionally, although the majority of published nomograms incorporate the same set of prognostic factors, it is unclear to what extent each of these factors remained as statistically significant predictors of BFFS across the different patient populations. In cases where only the nomogram is presented without a corresponding multivariable Cox regression model, there is no available mechanism to determine the significance level of a prognostic factor, only the relative importance based on the length of the predictor scales. For instance, with nomograms presented as a series of dichotomous prognostic factors, visual comparisons between adjacent prognostic factors can provide a reliable means to determine the relative importance of each and enable the observer to sort the variables in order of ascending or descending importance.

This analysis demonstrated that the use and duration of ADT administration had impacts on 5-year BFFS for both treatment cohorts. For LDR brachytherapy, the use of ADT (HR: 0.53; 95% CI: 0.37-0.76, p<0.001) was shown to have a protective impact within the first 12 months following treatment, as shown in Figure 2. Given the relatively low number of patients in the LDR brachytherapy cohort receiving ADT for one year or beyond (3.7%), questions still remain regarding the magnitude of benefit of prescribing ADT beyond one year following LDR brachytherapy, which could be addressed in the context of future clinical studies. For EBRT, increasing duration of ADT administration was found to provide a modest protective effect without a time constraint contrary to LDR brachytherapy (HR: 0.99, 95% CI: 0.97-1.00, p=0.092) and is consistent with previously published comparisons of EBRT versus EBRT + ADT [51-52]. Although the nomogram developed for the EBRT cohort incorporates a wide duration for ADT use, this is reflective of a comparatively more heterogeneous patient population with only 18% and 8% receiving ADT for at least two and three years, respectively.

The current study reports on retrospectively collected data from a variety of Canadian institutions, which may not be entirely representative of the greater Canadian population. Although the ProCaRS database underwent extensive quality assurances to improve data quality and accuracy as reported previously [43-44], heterogeneity in data collection procedures across institutions may still be present. Changes in treatment delivery and data entry may have occurred during the data collection period. Additionally, the database contains a wide range of parameters, including prognostic factors, treatment details, and survival-based outcomes; however, toxicity, comorbidity, and other important prognostic factors were not collected and were thus unavailable to assist in explaining the observations reported in the current study. The ability to evaluate long-term outcomes was also limited due to the few numbers of patients with observed follow-up durations in excess of 10 years, which factored into the decision to only report on biochemical outcomes. 

Future publications will focus on updates to the proposed nomograms and explore OS outcomes once sufficient follow-up data has been collected. In order to perform extensive multivariable regression analyses required for nomogram development, the final reported nomograms are reflective of only those patients with complete data for all variables under investigation. As a result, positive core biopsy information was only reported in univariable analysis procedures due to elevated rates of missing data. Patients receiving HDR-brachytherapy, plus EBRT, as reported in previous work were not examined due to fewer numbers of eligible patients available for analysis [43-44]. Although clinically useful, patients receiving EBRT were not examined separately according GUROC intermediate- versus high-risk classification given the insufficient statistical power. Future work examining a more extensive range of prognostic factors and comorbidities would provide a useful context for the presented work.

## Conclusions

This investigation developed and validated a set of clinical nomograms examining 5-year biochemical failure-free survival for patients with localized prostate cancer receiving either low-dose rate brachytherapy or conventionally fractionated external beam radiation therapy. This work is an extension of a Canadian multi-institutional initiative directed at the improvement of existing risk stratification tools. Future work should be directed at examining the role of additional prognostic factors, comorbidities, and toxicity data in predicting survival-based outcomes.
